# Effects of whole-body vibration training on physical function, bone and muscle mass in adolescents and young adults with cerebral palsy

**DOI:** 10.1038/srep22518

**Published:** 2016-03-03

**Authors:** Silmara Gusso, Craig F Munns, Patrícia Colle, José G B Derraik, Janene B Biggs, Wayne S Cutfield, Paul L Hofman

**Affiliations:** 1 Liggins Institute, University of Auckland, Auckland, New Zealand; 2 Endocrinology Department, The Children’s Hospital at Westmead, Sydney, Australia

## Abstract

We performed a clinical trial on the effects of whole-body vibration training (WBVT) on muscle function and bone health of adolescents and young adults with cerebral palsy. Forty participants (11.3–20.8 years) with mild to moderate cerebral palsy (GMFCS II–III) underwent 20-week WBVT on a vibration plate for 9 minutes/day 4 times/week at 20 Hz (without controls). Assessments included 6-minute walk test, whole-body DXA, lower leg pQCT scans, and muscle function (force plate). Twenty weeks of WBVT were associated with increased lean mass in the total body (+770 g; p = 0.0003), trunk (+410 g; p = 0.004), and lower limbs (+240 g; p = 0.012). Bone mineral content increased in total body (+48 g; p = 0.0001), lumbar spine (+2.7 g; p = 0.0003), and lower limbs (+13 g; p < 0.0001). Similarly, bone mineral density increased in total body (+0.008 g/cm^2^; p = 0.013), lumbar spine (+0.014 g/cm^2^; p = 0.003), and lower limbs (+0.023 g/cm^2^; p < 0.0001). Participants reduced the time taken to perform the chair test, and improved the distance walked in the 6-minute walk test by 11% and 35% for those with GMFCS II and III, respectively. WBVT was associated with increases in muscle mass and bone mass and density, and improved mobility of adolescents and young adults with cerebral palsy.

Cerebral palsy (CP) encompasses a number of non-progressive encephalopathies of varied aetiology. It usually presents at birth or in early infancy, and is the most common cause of physical disability in childhood with a prevalence of 2 per 1000 children^1^. The common features of the condition are impairment in muscle function, reduced muscle and bone mass, as well as varying degrees of impaired mobility^2^.

The reduced bone mass due to abnormal bone development is an adverse consequence that persists into adult life^2^,^3^. Up to 50% of children with CP will experience a fragility fracture, with an annualized incidence of 7%^4^. While the aetiology of low bone mass and density with increased fracture risk are multifactorial, reduced muscle mass and mobility are important factors^3^. To date, the management of bone health in CP has focussed on treatment of established bone fragility with bisphosphonates, rather than prevention of bone fragility through optimisation of physical therapy^5^,^6^,^7^.

With limited evidence on non-pharmaceutical treatments for CP, the use of whole-body vibration training (WBVT) has recently been investigated. Preliminary studies using WBVT in children with reduced mobility and/or decreased bone mass were associated with an improvement in both lower limb muscle function and bone mass^8^,^9^,^10^,^11^. A recent systematic review and meta-analysis examined the very limited data on this group, which covered only 6 studies and a total of 176 children^12^. These studies in children with CP have been relatively small or have been carried out on heterogeneous group of participants that makes interpretation of findings difficult. In addition, only one study has specifically examined the effects of WBVT as a monotherapy in children with CP^12^. Further, no studies have specifically examined the effects of WBVT on both bone and muscle in adolescents with CP. As a result, there is a need for robust data from clinical trials employing WBVT as a possible treatment for CP. By maintaining muscle mass and bone mineral accrual during growth, WBVT could maximise mobility and bone strength into adult life. Thus, in this clinical trial we aimed to determine the effects of 20 weeks of WBVT on the muscle and bone health of adolescents and young adults with mild to moderate CP.

## Methods

### Ethics

Ethics approval was granted by the Northern X Regional Ethics Committee. Written informed consent was obtained from all parents or guardians, as well as verbal or written consent from all able participants. This study was performed in accordance with all appropriate institutional and international guidelines and regulations for medical research, in line with the principles of the Declaration of Helsinki. The trial was registered (5^th^ May 2011) at the Australian New Zealand Clinical Trials Registry (ACTRN12611000465954).

### Participants

Adolescents and young adults with CP in the Auckland region of New Zealand were recruited by contacting the schools that cater for them with appropriate physiotherapy services, as well as via Auckland District Health Board and Counties Manukau District Health Board clinics. After establishing contact with families, schools and the respective physiotherapists, parents of potential participants were contacted via telephone, and their sons or daughters with CP were invited to the study. All recruited participants had mild to moderate CP, i.e. Level II or III disabilities as per the Gross Motor Function Classification System (GMFCS) for CP^13^. Exclusion criteria included having had a fracture within 8 weeks of enrolment, pregnancy, acute thrombosis, muscle or tendon inflammation, nephrolithiasis, discopathy, or arthritis. Other exclusion criteria were the use of anabolic agents, glucocorticoids (other than asthma inhalers), bisphosphonates or growth hormone. In addition, potential participants were also excluded if they displayed cognitive or physical impairment sufficiently severe that would hinder fulfilment of study protocol or clinical assessments. Serum 25-hydroxyvitamin D levels were obtained to ensure participants were not vitamin D deficient. No participants received botulinum toxin injection during the study or in the 3 months preceding its commencement.

### Trial design

The study was originally designed and registered as a randomized controlled trial. However, as a result of the likely benefits of WBVT, parents of all potential participants were adamant that they wanted their sons/daughters to undergo the actual intervention. Thus, the study was changed to a clinical trial without a control group, comparing baseline vs post-treatment outcomes within subjects.

### Training protocol

WBVT was performed on the Galileo Basic vibration plate (Novotec Medical, Pforzheim, Germany). Each session consisted of three 3-minute bouts of training, with a 3-minute rest between them. Sessions were performed four times a week, over a 20-week period. Participants started with sessions of three 1-minute bouts at 12 Hz, and both intensity and duration were gradually increased according to the response of individual participants. However, by the end of week 4, all participants were training at the prescribed protocol of 3 sets of 3 minutes at 20 Hz and 1 mm amplitude, four times a week. Majority of participants were training for 2 minutes at 15 Hz by the end of week 1, for 3 minutes at 16 Hz by week 2, for 3 minutes at 18 Hz by week 3, and for 3 minutes at 20 Hz by the end of week 4. Training intensity was maintained at 20 Hz for the remainder of the intervention. Participants stood barefoot on the plate with feet apart (position 2–3) and in parallel, with knees slightly bent for the duration of training. An adjustable metal frame was used as an aid for those participants who had difficulty in standing on the plate over the course of training.

The participants’ hands were mostly free during the training sessions. The frame was used in the beginning of the study until participants built up confidence and were able to safely support themselves during the vibration training. However, for the few participants with poorer balance (<10%), the frames were maintained at all times, so that it could be used to safely regain balance when necessary.

Training sessions were performed either at school or in the home environment to suit individual families. Sessions at school were supervised by a member of our research team, while parents/caregivers supervised home sessions. Participants were asked to maintain a training diary to monitor compliance with study protocol. Data recorded in the diary included the date, intensity and duration of training, as well as any comments regarding adverse events, tiredness, or pain. A research team member also supervised the participants performing training at home once a week, in order to monitor progress and provide feedback/support for parents or caregivers.

### Clinical assessments

All clinical assessments were performed at the Maurice and Agnes Paykel Clinical Research Unit (Liggins Institute, University of Auckland). Children’s heights were measured using a Harpenden stadiometer. Body mass, body composition, and skeletal data were obtained using whole-body dual-energy X-ray absorptiometry (DXA, Lunar Prodigy 2000, General Electric, Madison, WI, USA). DXA scans were also performed at the lumbar spine (L1–L4, in anterior-posterior view) and dual femur areas.

In addition, peripheral quantitative computed tomography (pQCT) (Stratec Medizintechnik GmbH, Pforzheim, Germany) was used to obtain cross-sectional measurements of tibial bone mass and calf muscle. Measurements were obtained at 4%, 20% and 50% sites along the tibia.

Physical function was assessed by the 6-minute walk test^14^. In brief, participants were asked to walk as fast as possible for exactly 6 minutes, with the total distance covered and the time taken to reach individual milestones recorded. Blood pressure and pulse rate were measured in a sitting position at rest prior to the start of test and immediately after completion. Blood pressure was measured using a standard mercury sphygmomanometer (Dinamap ProCare 100, GE Healthcare, Freiburg, Germany), with an appropriately-sized cuff on the non-dominant arm.

Muscle function was assessed using the Leonardo Mechanography Ground Reaction Force Plate (Novotec Medical, Pforzheim, Germany). Assessments included:

- the chair rising test – performed with a specially-designed seat (Novotec Medical) placed on the plate, with participants standing up and sitting down three times as fast as possible;
- single two-leg jump – jumping as high as possible using both legs and landing on the forefoot;
- balance test – standing still on the plate for 10 seconds.

All assessments were performed three times. The first assessment aimed to familiarize the participants with the test. The average of the last two assessments was used as the final result.

Participants and their families were asked to report to investigators any adverse events that could possibly be associated with the WBVT.

### Health-related quality of life

Health-related quality of life was assessed as per the Cerebral Palsy Quality of Life (CP QOL) questionnaire v.3 15. Specific questionnaires are applied to caregivers (89 questions) and participants (72 questions) that cover a number of different domains: communication and physical health, feelings about functioning, general well-being and participation, pain and impact of disability, school well-being, social well-being, as well as access to services and family health (for caregivers only). A global score was obtained by averaging all scores for a given individual. Questions were rated on a scale of 1–9 and then transformed into scores that ranged from 0 to 100. Data were only included for participants or caregivers who had completed the questionnaire both at baseline and at the end of the treatment period.

### Statistical analyses

Associations between demographic parameters and compliance were assessed with Pearson’s correlation coefficient or one-way ANOVA. Treatment effects were assessed using random effects mixed models with repeated measures, while adjusting for GMFCS level and the baseline value of the outcome response (i.e. baseline data were included in the model as covariates). Regression models were also run to evaluate the potential effects of compliance, gender, and place of training on treatment outcomes. Data were examined for a normal distribution using the Anderson-Darling test, and where necessary, data were log-transformed to approximate normality. Parametric and non-parametric tests were used for certain quality of life parameters with a skewed distribution, but as the results were similar, we report just the parametric results. Only observed data were used, and there was no imputation of missing values. Univariate analyses were carried out in Minitab v.16 (Pennsylvania State University, State College, PA, USA) and multivariate analyses in SAS v.9.3 (SAS Institute, Cary, NC, USA). All statistical tests were two-tailed and significance level maintained at 5%. Data are expressed as means ± standard errors of the mean.

## Results

A total of 40 participants took part in the study, including 34 GMFCS II and 6 GMFCS III. Participants had a mean age of 16.2 years (SD = 2.1; range 11.3–20.8 years), comprising 23 males and 17 females. Compliance with prescribed study protocol was variable but relatively high overall at 74%; 28% of participants performed more than 90% of prescribed sessions, and most (80%) completed ≥50%. Note that an uneven number of participants completed the individual assessments, and the respective *n* is provided in Tables 1 and 2.

Post-training, participants displayed increased lean mass at the total body level (+770 g (+2.1%); p = 0.0003), in the trunk (+410 g (+2.4%); p = 0.004), and in the lower limbs (+240 g (+2.2%); p = 0.012), without any changes in fat mass (Table 1). The increase in leg muscle mass was confirmed by pQCT data, with greater leg muscle area observed at 20% (+5.6%; p = 0.0006) and 50% (+3.8%; p = 0.0009) along the tibia (Table 1).

There were also consistent improvements in bone mass measured by DXA, with bone mineral content increasing in total body (+2.3%; p = 0.0001), lumbar spine (+5.1%; p = 0.0003), and lower limbs (+2.0%; p < 0.0001) (Table 1). Similarly, bone mineral density also increased in total body (+0.8%; p = 0.013), lumbar spine (+1.3%; p = 0.003), and lower limbs (+2.2%; p < 0.0001) (Table 1).

Functional tests in the Leonardo platform showed some improvement. Participants reduced the time taken to perform the chair test by 1.5 seconds (p = 0.0004) and tended to have increased power (+0.4 kW; p = 0.060) (Table 1). However, there was no evidence of improvements in the jump and balance tests (Table 1).

### 6-minute walk test

**Figure 1:**
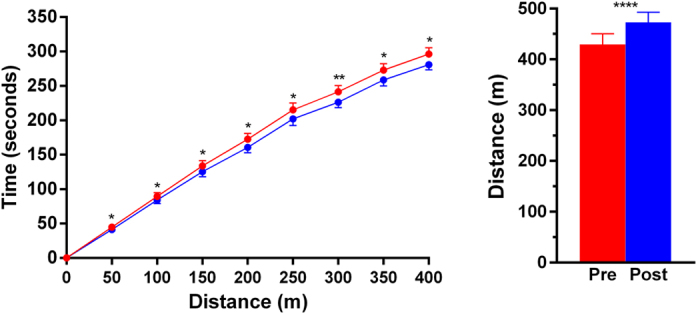
Performance of GMFCS II participants in the 6-minute walk test prior to (red) and after (blue) 20 weeks of whole-body vibration training. Note that 34 participants started the tests, but only 21 and 22 reached the 400-metre mark at baseline and post-training, respectively. Data are means ± standard errors of the mean. *p < 0.05, **p < 0.01, and ****p < 0.0001 for baseline vs post-training.

GMFCS II participants walked 44 meters further during the 6-minute walk test after WBVT (Fig. 1), with an average 11% improvement (p = 0.0001). These participants walked faster post-training, with sustained reductions in the time taken to reach individual milestones throughout the 6-minute test (Fig. 1).

**Figure 2:**
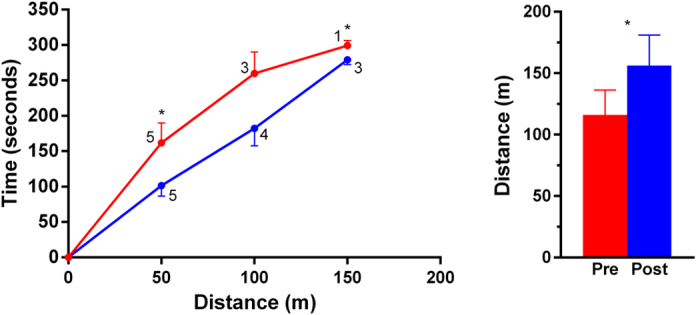
Performance of GMFCS III participants in the 6-minute walk test prior to (red) and after (blue) 20 weeks of whole-body vibration training. Six subjects started the tests, and the number of participants reaching a particular milestone is shown in the figure. Data are means ± standard errors of the mean. *p < 0.05 for baseline vs post-training.

There was an even greater improvement in the 6-minute walk test among the six GMFCS III participants. They walked extra 40 metres on average, equating to a mean improvement of 35% (p < 0.0001), with three participants reaching the 150 metres milestone (compared to only one at baseline) (Fig. 2). GMFCS III participants also walked faster post-training (Fig. 2).

### Health-related quality of life

Compliance with the CP QOL questionnaire was poor and only 16 caregivers and 12 participants completed them both at baseline and post treatment. Note that amongst the 8 caregiver-participant pairs who answered all questionnaires, caregiver and participant scores were positively and highly correlated, with the exception of school well-being scores (data not shown).

Participants did not report any improvements in health-related quality of life (Table 2). However, caregivers reported that they observed improvements in school well-being (p = 0.014) and general well-being and participation (p = 0.002), as well as a considerable decrease in the perceived pain and impact of disability (p < 0.0001) (Table 2). As a result, caregivers reported a global improvement in quality of life (p = 0.037; Table 2).

### Potential confounders

There was no evidence that the level of compliance with study protocol affected study outcomes. There was also no evidence of sex-specific effects of vibration training, or of any differences in outcomes between those who trained at school or in the home environment (data not shown).

Compliance was not affected by gender (males 71% vs females 77%; p = 0.53) or place of training (home 77% vs school 69%; p = 0.36). However, increasing age was correlated with poorer compliance with study protocol (r = −0.36; p = 0.025).

### Adverse events

There were no recorded adverse events in association with WBVT. However, the parents of four participants who were afflicted by constipation reported improvements in bowel function while undergoing WBVT.

## Discussion

There has been limited research on the effects of WBVT in adolescents with CP. Previous research findings were diverse due to the mixed levels of gross motor function of study participants, length of training, heterogeneous aetiology etiology, and the various outcomes investigated. This is the first study to investigate the impact of prolonged WBVT on both muscle function and bone health in adolescents and young adults with mild to moderate CP.

The present study showed significant improvements on mobility, as seen by an increase in the distance walked during the 6-minute walk test and by improvements on functional tasks (e.g. rising from a chair) following WBVT. Notably, those with more significant functional impairment (GMFCS III) had greater improvements on mobility compared to those with milder functional impairment (GMFCS II). These findings are similar to the results from Ibrahim *et al*. who observed that 12 weeks of WBVT in 15 children (aged 8–12 years) with spastic diplegic CP improved the walking speed during the 6-minute walk test^16^. Mobility is an important aspect of motor performance, being positively associated with health-related quality of life particularly in physical domains^17^,^18^,^19^. Thus, therapies that increase mobility have the potential to greatly improve the quality of life and engagement in activities among children and adults with CP. In this study, in addition to the above-described improvements on mobility, parents reported improvements in quality of life of participants.

DXA data showed an overall increase in lean mass following WBVT, with improvements in both trunk and lower limbs. Moreover, the pQCT data confirmed this increase in muscle mass in study participants as seen by greater muscle area at the lower leg. These results are consistent with previous research using combined therapies (including WBVT) that showed increases in muscle mass of approximately 3%^9^. Our results are important, as decreased muscle mass is a considerable problem for children and adolescents with CP, and strategies to increase muscle mass in these patients are difficult to implement. In addition, inadequate bone development and osteoporosis in children with CP are primarily due to reduced muscle mass and poor mobility, which seem to be ameliorated by the improvements observed with WBVT.

WBVT provided by the Galileo plate is characterized by seesaw movements that stimulate a moving pattern similar to human gait^20^. These result in the activation of proprioreceptive spinal circuits, thus leading to compensatory rhythmic muscle contractions in the lower limbs and trunk^20^. Our participants not only displayed improvements in muscle mass, but also improvements in bone mineral content and density ranging from 1 to 5%. The increase in bone mass was probably due to the effect of muscle action on bone during WBVT, combined with the effects of the observed improved mobility. Thus, our findings fit well with the mechanostat theory, in which mechanical loading (in this case muscle contractions generated by the vibration platform and improved mobility) resulted in positive effects on bone, such as an increase in bone mass. Note that our results were similar to those observed by Stark *et al*. after 6 months of combined therapy in a group of 78 children with GMFCS I to V, who reported 2.3% and 5.7% increases in total bone mineral density and content, respectively^9^. However, their intervention involved not only WBVT, but also other forms of physiotherapy, resistance training, and treadmill workout, which made it impossible to identify the specific effects of WBVT. Nonetheless, Wren *et al*. looked at the impact of daily vibration training alone on bone health of children with CP aged 6 to 12 years, observing improvements in cortical bone area in comparison to a regimen of simply standing on the floor^8^.

A limitation of our study was the uneven number of participants that were able to successfully conclude all the necessary assessments. Data for some participants were also of poor quality, as these subjects were simply unable to keep their limbs still during scanning. Thus, the decreased data available for analyses likely reduced the power of our study to detect treatment effects. This was also the case for the health-related quality of life questionnaires, for which completion rates were poor. In addition, although our study involved a relatively homogeneous group of patients with CP, there were only six participants with GMFCS III, which limit our ability to extrapolate findings to those with more severe impairments in gross motor function. Lastly, we also did not have a control group. However, this is the first study on the effects of WBVT in adolescents with CP, which involved comprehensive assessments of muscle function and bone health.

In conclusion, WBVT was associated with increases in muscle mass and bone mass and density, while improving mobility in adolescents and young adults with mild to moderate cerebral palsy. Importantly, parents also perceived improvements in health-related quality of life with WBVT among study participants. Nonetheless, it is still unclear whether the observed positive effects persist after WBVT ceases. Thus, the efficacy of WBVT for the long-term management of cerebral palsy requires further investigation in larger and longer trials.

## Additional Information

**How to cite this article**: Gusso, S. *et al*. Effects of whole-body vibration training on physical function, bone and muscle mass in adolescents and young adults with cerebral palsy. *Sci. Rep.* **6**, 22518; doi: 10.1038/srep22518 (2016).

## Figures and Tables

**Table 1:** Study outcomes among 40 participants with cerebral palsy at baseline and after a 20-week training period on the Galileo vibration plate.

			**Baseline**	**Post-training**	**p-value**
DXA (n = 39)	Weight	(kg)	53.13 ± 2.74	53.94 ± 2.66	**0.013**
	BMI	(kg/m^2^)	21.93 ± 0.79	21.97 ± 0.76	0.79
	Fat mass	Total (kg)	15.22 ± 1.87	15.23 ± 1.85	0.97
	Lean mass	Total (kg)	36.00 ± 1.98	36.77 ± 1.96	**0.0003**
		Trunk (kg)	17.27 ± 0.95	17.68 ± 0.94	**0.004**
		Lower limbs (kg)	10.74 ± 0.65	10.98 ± 0.63	**0.012**
	BMC	Total (g)	2097 ± 120	2145 ± 120	**0.0001**
		Lumbar spine (g)	51.85 ± 3.39	54.51 ± 3.33	**0.0003**
		Lower limbs (g)	642 ± 39	655 ± 38	**<0.0001**
		Femur (g)	25.43 ± 1.45	25.68 ± 1.43	0.20
	BMD	Total (g/cm^2^)	1.060 ± 0.025	1.068 ± 0.024	**0.013**
		Lumbar spine (g/cm^2^)	1.095 ± 0.042	1.109 ± 0.042	**0.003**
		Lower limbs (g/cm^2^)	1.048 ± 0.033	1.071 ± 0.033	**<0.0001**
		Femur (g/cm^2^)	0.954 ± 0.034	0.969 ± 0.035	**0.034**
pQCT (n = 26)	BMD	Tibia 20% (mg/cm^3^)	687 ± 34	686 ± 34	0.77
		Tibia 50% (mg/cm^3^)	754 ± 28	755 ± 29	0.82
	SSIp	Tibia 20% (mm^3^)	854 ± 117	863 ± 116	0.11
		Tibia 50% (mm^3^)	1274 ± 178	1280 ± 177	0.83
	Muscle area	Leg 20% (mm^2^)	1442 ± 126	1523 ± 123	**0.0006**
		Leg 50% (mm^2^)	3538 ± 396	3672 ± 390	**0.0009**
Functional tests	Chair test (n = 37)	Velocity (m/s)	0.56 ± 0.07	0.58 ± 0.07	0.57
		Time (s)	8.54 ± 0.82	7.03 ± 0.65	**0.0004**
		Power (kW)	6.18 ± 0.78	6.57 ± 0.83	0.060
	Jump test (n = 29)	Jump height (m)	0.22 ± 0.02	0.25 ± 0.02	0.33
		Maximum power (kW)	1.40 ± 0.12	1.46 ± 0.12	0.16
	Balance test (n = 35)	Both legs area (cm^2^)	2.55 ± 0.42	2.27 ± 0.34	0.18

Data are means ± standard errors of the mean. P-values refer to the changes from baseline, and statistically significant results at p < 0.05 are shown in bold. DXA, whole-body dual-energy X-ray absorptiometry; BMC, bone mineral content; BMD, bone mineral density; BMI, body mass index; pQCT, peripheral quantitative computed tomography; SSIp, polar stress-strain index. Note that certain participants were not able to perform all assessments or the quality of the data gathered (particularly for the pQCT) was too low for inclusion in the analyses.

**Table 2:** Health-related quality of life as assessed by caregivers (n = 16) and self-reported by the participants (n = 12), according to the Cerebral Palsy Quality of Life questionnaire (CP QOL).

	**Domain**	**Baseline**	**Post-training**	**P-value**
Caregivers	Access to services	63 ± 5	67 ± 5	0.14
	Communication & physical health	69 ± 2	68 ± 2	0.40
	Family health	59 ± 2	60 ± 2	0.67
	Feelings about functioning	60 ± 4	60 ± 4	0.99
	General well-being and participation	66 ± 2	71 ± 2	**0.002**
	Pain and impact of disability	60 ± 6	76 ± 6	**<0.0001**
	School well-being	65 ± 3	70 ± 3	**0.014**
	Social well-being	83 ± 3	84 ± 3	0.58
	Global quality of life	66 ± 2	70 ± 2	**0.037**
Participant	Communication & physical health	78 ± 7	78 ± 7	0.87
	Feelings about functioning	55 ± 5	63 ± 5	0.06
	General well-being and participation	76 ± 8	77 ± 8	0.67
	Pain and impact of disability	94 ± 14	87 ± 14	0.10
	School well-being	77 ± 9	78 ± 9	0.65
	Social well-being	90 ± 4	85 ± 4	0.16
	Global quality of life	78 ± 7	78 ± 7	0.88

Data are transformed scores (range 0–100) and are expressed as means ± standard errors of the mean. For all domains higher values mean better outcomes. P-values refer to the changes from baseline, and statistically significant results at p < 0.05 are shown in bold.
